# Source and regulation of flux variability in *Escherichia coli*

**DOI:** 10.1186/1752-0509-8-67

**Published:** 2014-06-14

**Authors:** Magdalena San Román, Héctor Cancela, Luis Acerenza

**Affiliations:** 1 Systems Biology Laboratory, Faculty of Sciences, Universidad de la República, Iguá 4225, Montevideo 11400, Uruguay; 2 Computer Science Institute, Faculty of Engineering, Universidad de la República, Montevideo, Uruguay

**Keywords:** Metabolic network, Flux variability, Metabolic flexibility, Physiological adaptation, Evolutionary adaptation, Systems biology

## Abstract

**Background:**

Metabolic responses are essential for the adaptation of microorganisms to changing environmental conditions. The repertoire of flux responses that the metabolic network can display in different external conditions may be quantified applying flux variability analysis to genome-scale metabolic reconstructions.

**Results:**

A procedure is developed to classify and quantify the sources of flux variability. We apply the procedure to the latest *Escherichia coli* metabolic reconstruction, in glucose minimal medium, with an additional constraint to account for the mechanism coordinating carbon and nitrogen utilization mediated by α-ketoglutarate. Flux variability can be decomposed into three components: internal, external and growth variability. Unexpectedly, growth variability is the only significant component of flux variability in the physiological ranges of glucose, oxygen and ammonia uptake rates. To obtain substantial increases in metabolic flexibility, *E. coli* must decrease growth rate to suboptimal values. This growth-flexibility trade-off gives a straightforward interpretation to recent work showing that most overall cell-to-cell flux variability in a population of *E. coli* can be attained sampling a small number of enzymes most likely to constrain cell growth. Importantly, it provides an explanation for the global reorganization occurring in metabolic networks during adaptations to environmental challenges. The calculations were repeated with a pathogenic strain and an old reconstruction of the commensal strain, having less than 50% of the reactions of the latest reconstruction, obtaining the same general conclusions.

**Conclusions:**

In *E. coli* growing on glucose, growth variability is the only significant component of flux variability for all physiological conditions explored. Increasing flux variability requires reducing growth to suboptimal values. The growth-flexibility trade-off operates in physiological and evolutionary adaptations, and provides an explanation for the global reorganization occurring during adaptations to environmental challenges. The results obtained do not rely on the knowledge of kinetic and regulatory details of the system and are highly robust to incomplete or incorrect knowledge of the reaction network.

## Background

The survival of microorganisms in changing environments is based on the ability of metabolic networks to show adaptive responses. Adaptive potential depends on the variety of responses that the organism can display and is limited by constraints in network’s stoichiometry, rate laws and regulation [1]. The greatest metabolic flexibility is achieved when kinetics and regulation are not limiting, adaptive responses being restricted by stoichiometric constraints only. This has been shown to be the case for the growth performance of *Escherichia coli* on glucose, reaching the optimal growth value calculated from a genome-scale *in silico* model [2]. On the other hand, growth on glycerol is suboptimal but, when placed under selective pressure, the population undergoes adaptive evolution to reach the predicted optimal growth [3].

Trade-offs between metabolic pathways may restrict growth to suboptimal values. In *Bacillus subtilis*, regulatory knockout mutants show improved biomass productivity, suggesting that the bacteria invest resources in anticipation of environmental conditions at the expense of optimal growth [4]. Data from gene expression levels in *E. coli* are consistent with setting up a suboptimal growth to increase metabolic flexibility [5]. Product formation may also compete with growth, what is relevant for bioprocess optimization in metabolic engineering [6]. The trade-off between catabolic functions and fitness was studied in a long term *E. coli* evolution experiment, showing that the decay of unused catabolic functions makes an important contribution to growth rate increase during evolution [7-9]. These trade-offs impose internal limitations on the flux responses that the organism can display. Changes in the composition of the external milieu may also mould the shape and size of the space of alternative solutions. As a result, the repertoire of flux responses may be described by a high dimensional polyhedron, whose shape and size is conditioned by environmental changes [10].

How the organism uses its metabolic capabilities to respond to environmental changes is still poorly understood. For instance, metabolic networks show extensive reorganization during adaptation to external perturbations, resulting in a rewiring of global network fluxes. In these global transitions, the expressions of hundreds or thousands of genes change by large factors [11-16]. A major challenge is to understand why global changes take place, even when the same adaptive response could be achieved with a small number of changes.

In the present work, we develop a procedure, combining flux variability analysis (FVA) [17] and flux balance analysis FBA [18], to analyze the sources of flux variability in different conditions. Flux variability is decomposed into three components, originated by three different sources: the intrinsic variation of the internal reactions, the variation of the exchange reactions and the variation of the flux to biomass production. The procedure is applied to the genome-scale reconstruction *iJO1366*[19], the latest and more comprehensive *E. coli* K-12 MG1655 metabolic reconstruction to date. The original model includes the stoichiometric constraints on the steady-state fluxes and the lower and upper bounds of fluxes under physiological conditions. Here, we introduce an additional constraint to account for the coordination of carbon and nitrogen utilization achieved with a regulatory mechanism, recently described in *E. coli*[20]. We change the input fluxes of glucose, ammonia and oxygen in the physiological range, studying the contribution of each source of variability in all conditions. A central result is that coordination of carbon and nitrogen utilization is sufficient to have growth variation as the only significant source of flux variability. The increase in metabolic flexibility requires decreasing growth to suboptimal values. This result indicates that the balance between the contradictory objectives of growth efficiency and metabolic flexibility could be modulated through the regulation of growth alone [21]. Our findings give a general interpretation to recent work showing that a large portion of the overall cell-to-cell variability in the flux patterns of *E. coli* may be explained by a small number of enzymes most likely to constrain cell growth [22]. In addition, they provide an explanation for the global network reorganization occurring during metabolic adaptations to environmental changes [11-16].

## Results

### Components of flux variability

Genome-scale *in silico* models represent the maximum metabolic capabilities of the organism [18]. They have two types of constraints: equations that balance reaction inputs and outputs at steady state and inequalities that impose the maximum and minimum allowable fluxes of the reactions. These constraints define the space of feasible flux solutions of the system. Kinetic and regulation constraints, not included in these models, impose additional limitations on metabolic capabilities (see also Additional file 1, section 1).

The volume of the space containing all flux solutions may be used as a measure of metabolic flexibility. When a change in the environmental conditions reduces the volume of feasible solutions, the number of ways to perform a given function is decreased, and metabolic responses become less flexible. To quantify changes in metabolic flexibility we use Δ, defined as [17,23]: $\Delta =\sum_{i=1}^{r}\left[\left(j_{i}^{\text{max}}-j_{i}^{\text{min}}\right)/\left(V_{i}^{\text{max}}-V_{i}^{\text{min}}\right)\right]/r$. $V_{i}^{\text{max}}$ and $V_{i}^{\text{min}}$ are the maximum and minimum flux values of reaction *i* in the reference conditions, and $j_{i}^{\text{max}}$ and $j_{i}^{\text{min}}$ are the same values under the conditions of interest (to be compared against the reference ones). The sum is performed over the *internal* reactions only. Δ quantifies how metabolic flexibility changes with respect to the reference conditions. For conditions more constrained than the reference ones, it always holds that 0 ≤ Δ < 1. If, in the more constrained conditions, the variability of only a small proportion of the reactions is decreased, the effect on the average would be very small, even when those rates were affected in a large extent. As a consequence, Δ values much smaller than one require that a large proportion of the reactions are significantly affected. For instance, Δ = 0.5 may represent that every reaction in the system looses 50% of its reference variability, that 50% of the reactions in the system loose all their variability or any intermediate situation. Therefore, Δ is particularly suited to quantify global changes in metabolic flexibility induced by different environmental conditions (see also Additional file 1, section 2).

Δ is calculated over the internal reactions only. However, the variability of the internal reactions may, in principle, come from three different sources: the intrinsic variation of the internal reactions, the variation of the external reactions and the variation of the growth rate. We call these sources: internal, external and growth variation, respectively.

**Figure 1:**
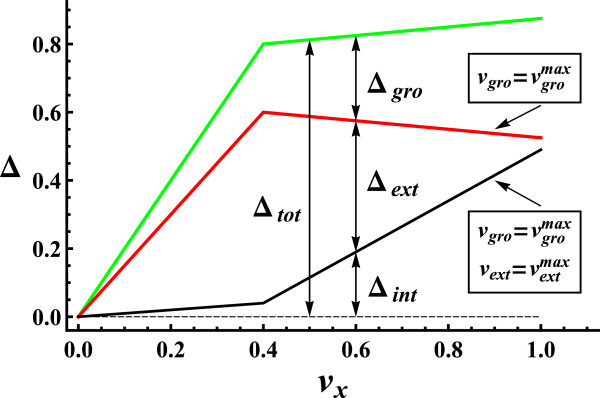
**Components of flux variability vs. uptake rate.***v*_*x*_ is the uptake rate of the external metabolite *x. Δ*_*int*_ (black line) is the internal variability, obtained under the constraints: $v_{\text{gro}}=v_{\text{gro}}^{\text{max}}$ and $v_{\text{ext}}=v_{\text{ext}}^{\text{max}}$. *Δ*_*int*_ + *Δ*_*ext*_ (red line) is the internal and external variability, obtained under the constraint: $v_{\text{gro}}=v_{\text{gro}}^{\text{max}}$. *Δ*_*tot*_ = *Δ*_*int*_ + *Δ*_*ext*_ + *Δ*_*gro*_ (green line) is the total variability, including the internal, external and growth components acting together.

Next we develop a procedure to determine the quantitative importance of each of the three sources of flux variability. We represent the rates of the internal, external and growth reactions by *v*_*int*_, *v*_*ext*_ and *v*_*gro*_. For a given set of conditions, $v_{\text{gro}}^{\text{max}}$ is the maximum growth rate and $v_{\text{ext}}^{\text{max}}$ the values of the external rates for the optimal growth solution. The contribution of internal variation to flux variability is the value Δ_*int*_ obtained under the constraints: $v_{\text{gro}}=v_{\text{gro}}^{\text{max}}$ and $v_{\text{ext}}=v_{\text{ext}}^{\text{max}}$ (see schematic representation of Figure 1). If we fix *v*_*gro*_ only ($v_{\text{gro}}=v_{\text{gro}}^{\text{max}}$), the Δ value resulting from the calculation (Δ_*int* + *ext*_) includes the effects of internal and external variation, acting together. Subtracting Δ_*int*_ from Δ_*int* + *ext*_, we obtain the additional variability obtained from external variation, Δ_*ext*_ (Figure 1). Finally, growth variation may further increase flux variability. Releasing *v*_*gro*_ (and *v*_*ext*_), the variability obtained, Δ_*tot*_, results from the simultaneous action of the three sources: internal, external and growth variation. The contribution made by growth variation to flux variability, Δ_*gro*_ , is the difference between Δ_*tot*_ and Δ_*int* + *ext*_ (Figure 1). Internal (Δ_*int*_), external (Δ_*ext*_), growth (Δ_*gro*_) and total (Δ_*tot*_) flux variability, fulfill: Δ_*tot*_ = Δ_*int*_ + Δ_*ext*_ + Δ_*gro*_. Note that the steady-state condition imposes that fixing only $v_{\text{ext}}=v_{\text{ext}}^{\text{max}}$ gives the same Δ value as fixing, simultaneously, $v_{\text{gro}}=v_{\text{gro}}^{\text{max}}$ and $v_{\text{ext}}=v_{\text{ext}}^{\text{max}}$. Note also that there may be alternative solutions to the patterns of external rates fulfilling optimal growth ($v_{\text{ext}}^{\text{max}}$). In this case, Δ_*int*_ and Δ_*ext*_ may change but their sum (Δ_*int* + *ext*_) and Δ_*gro*_ remain unaltered.

The reference conditions used in this work to calculate Δ are those of the glucose minimal medium, described in Additional file 2. Calculations are performed in aerobic ($v_{O_{2}}>0$) and anaerobic ($v_{O_{2}}=0$) conditions. Δ_*tot*_ for the glucose minimal medium in aerobic conditions is, by definition, equal to 1. Imposing $v_{O_{2}}=0$ we obtain: Δ_*tot*_ = 0.207, which is the maximum variability that can be achieved for the glucose minimal medium in anaerobic conditions. Thus, the absence of oxygen reduces the variability to approximately 20% of the reference value.

Δ may be used to estimate the metabolic flexibility that an organism can display during physiological and evolutionary adaptations. For this purpose, we use the stoichiometry matrix representing the organism (Additional file 1, section 1). Note that evolutionary processes resulting in new catalytic activities, which were not present in the original organism, change the stoichiometry matrix. In this type of evolutionary adaptations, metabolic flexibility, calculated with the stoichiometry matrices of the evolved organisms, may change significantly.

### Flux variability vs. uptake rates

Changes in the external milieu may affect flux variability. Next, we study how flux variability depends on the uptake rates of glucose, oxygen and ammonia. To perform the analysis we calculate Δ for different fixed values of the uptake rates. For each of these values, the three components of flux variability (Δ_*int*_, Δ_*ext*_ and Δ_*gro*_, Figure 1) are calculated.

**Figure 2:**
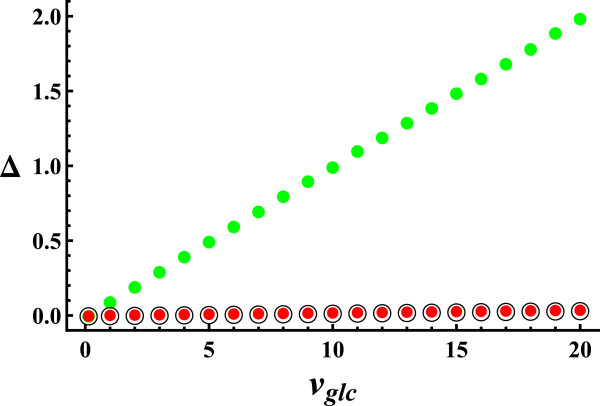
**Components of flux variability vs. glucose uptake in aerobic conditions.** Black circle represents *Δ*_*int*_, red circle represents *Δ*_*int*_ + *Δ*_*ext*_ and green circle represents *Δ*_*tot*_ = *Δ*_*int*_ + *Δ*_*ext*_ + *Δ*_*gro*_. *Δ*_*gro*_ is the only significant component of flux variability, *Δ*_*int*_ and *Δ*_*ext*_ having negligible values for all the range of glucose uptakes studied (the physiological values of glucose uptake are between 0 and 10).

In Figure 2, we represent the components of flux variability (sketched in Figure 1) as a function of glucose uptake, in aerobic conditions. The curves start at *v*_*Glc*_ = 0.13, which is the minimum value fulfilling the flux of ATP consumption for cell maintenance. The plot extends beyond the upper bound of glucose uptake ($v_{\text{Glc}}^{\mathrm{ub}}=10$, Additional file 2), in order to test if the conclusions obtained depend on how the physiological range is defined. We see that Δ_*int*_ and Δ_*ext*_ are negligible for all the range studied, Δ_*gro*_ being the only important component of flux variability. The increase in Δ_*gro*_ with glucose uptake is consistent with the increase in maximum growth observed with glucose uptake (see Additional file 3). Similar conclusions are obtained for the components of flux variability as a function of glucose uptake in anaerobic conditions (see Additional file 4).

**Figure 3:**
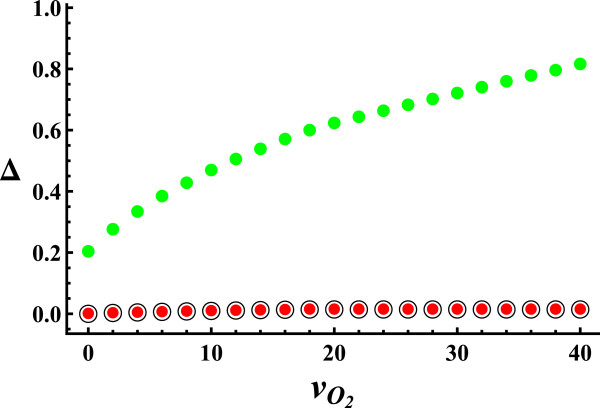
**Components of flux variability vs. oxygen uptake.** Black circle represents *Δ*_*int*_, red circle represents *Δ*_*int*_ + *Δ*_*ext*_ and green circle represents *Δ*_*tot*_ = *Δ*_*int*_ + *Δ*_*ext*_ + *Δ*_*gro*_. *Δ*_*gro*_ is the only significant component of flux variability, *Δ*_*int*_ and *Δ*_*ext*_ having negligible values for all the range of oxygen uptakes studied.

In the range of oxygen uptake studied ($0<v_{O_{2}}<40$, Figure 3), the components of flux variability show the same behavior as for glucose uptake, i.e. flux variability increases with oxygen uptake, Δ_*gro*_ being the only significant component of flux variability.

**Figure 4:**
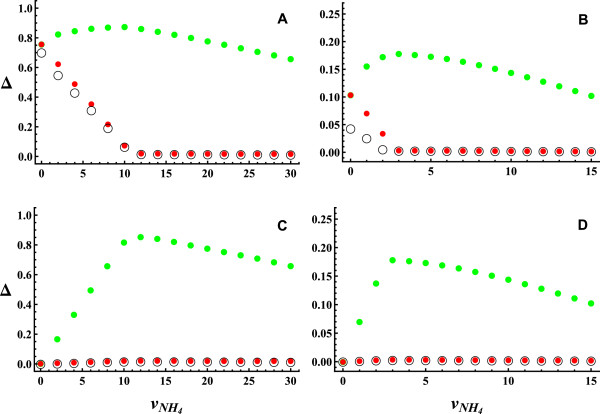
**Components of flux variability vs. ammonia uptake.** Black circle represents *Δ*_*int*_, red circle represents *Δ*_*int*_ + *Δ*_*ext*_ and green circle represents *Δ*_*tot*_ = *Δ*_*int*_ + *Δ*_*ext*_ + *Δ*_*gro*_. **(A)** Aerobic conditions without coordination of carbon and nitrogen uptake. **(B)** Anaerobic conditions without coordination of carbon and nitrogen uptake. **(C)** Aerobic conditions with coordination of carbon and nitrogen uptake. **(D)** Anaerobic conditions with coordination of carbon and nitrogen uptake. The contribution of *Δ*_*int*_ and *Δ*_*ext*_ to flux variability, at low ammonia uptakes, disappears when the coordination of carbon and nitrogen utilization is imposed. Under coordination of carbon and nitrogen utilization, *Δ*_*gro*_ is the only significant component of flux variability.

In Figure 4A, we represent the components of flux variability as a function of ammonia uptake in aerobic conditions. For ammonia uptakes greater than the value corresponding to optimum growth (i.e. $v_{NH_{4}}>10.6$), the only component contributing to flux variability is Δ_*gro*_. For $v_{NH_{4}}<10.6$, Δ_*int*_ and Δ_*ext*_ also make significant contributions to flux variability, the contribution of Δ_*int*_ being quantitatively more important than the contribution of Δ_*ext*_. A similar pattern is obtained in anaerobic conditions (Figure 4B). The difference is that in the interval where Δ_*int*_ and Δ_*ext*_ make significant contributions to flux variability, Δ_*ext*_ predominates over Δ_*int*_. This is the only case encountered where there is a significant contribution of internal and external variability to flux variability in the physiological range of uptake rates.

### Coordination of carbon and nitrogen utilization

In the previous calculations, two types of constraints were included: the stoichiometric constraints on the steady-state fluxes and lower and upper bounds for these fluxes. Here, we introduce an additional constraint, to account for the mechanism coordinating carbon and nitrogen utilization in *E. coli*[20]. As we shall see, this physiological constraint further reduces the space of alternative solutions, affecting flux variability and its components.

Glucose uptake in *E. coli* is regulated in response to perturbations in nitrogen availability. Under nitrogen limitation conditions, α-ketoglutarate accumulates, blocking glucose uptake by inhibiting the first step of the phosphotransferase system. Simple differential-equations simulations confirm that this inhibition is sufficient to match glucose consumption to the nitrogen-controlled growth rate [20]. To determine the effect of this mechanism on the variability patterns, when nitrogen uptake is changed, we use the following procedure. For each fixed value of the rate of ammonia uptake ($v_{NH_{4}}$), first, *v*_*gro*_ is maximized to obtain $v_{\text{gro}}^{\text{max}}$ and, second, *v*_*glc*_ is minimized subject to $v_{\text{gro}}=v_{\text{gro}}^{\text{max}}$. In this way, glucose uptake is coordinated with nitrogen uptake, taking the minimum value required to obtain the maximum growth which can be achieved for the fixed value of $v_{NH_{4}}$ considered ($v_{\text{Glc}}^{\text{min}}$). Δ is calculated fixing *v*_*glc*_ to $v_{\text{Glc}}^{\text{min}}$. In Figures 4C and D, we represent Δ vs. $v_{NH_{4}}$, under coordination of carbon and nitrogen utilization, in aerobic and anaerobic conditions, respectively. The contributions of Δ_*int*_ and Δ_*ext*_ are reduced to almost zero, Δ_*gro*_ being the only significant contribution to flux variability.

In summary, introducing the coordination of carbon and nitrogen utilization [20] as an additional physiological constraint, growth variability is the only significant component of flux variability in the physiological range of glucose, oxygen and ammonia uptakes.

### Flux variability vs. growth rate

The analysis performed shows that growth variability is the only significant component of flux variability in all conditions studied. In this section, we analyze how flux variability depends on the values at which the upper and lower bounds of the growth rate are set.

**Figure 5:**
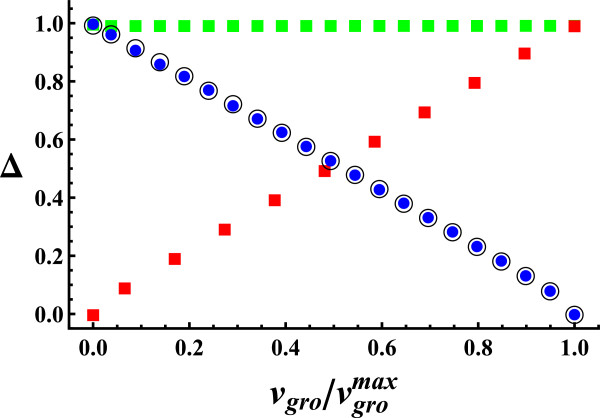
**Total variability vs. growth rate, in aerobic conditions.** Red square corresponds to fixing glucose uptake at different values between 0 and 10 (keeping growth free), green square corresponds to fixing growth upper bound (keeping growth lower bound at zero and glucose uptake free), black circle corresponds to fixing growth lower bound (keeping growth upper bound at maximum growth and glucose uptake free) and blue circle corresponds to fixing upper and lower growth bounds at the same value (keeping glucose uptake free). Red square curve shows that growth limitation by glucose uptake produces an approximately proportional reduction in flux variability. Green square curve indicates that fixing growth upper bound has a negligible effect on flux variability. Black circle and blue circle curves show that fixing growth lower bound or both bounds have the same effect. Therefore, high flux variability requires that glucose uptake is high, so that the organism can grow at a potentially high rate, and that growth rate can be regulated to much lower values than the potential maximum growth.

Fixing glucose uptake constrains the value of Δ (Figure 2) and maximum growth (see Additional file 3). To see how Δ is related to the value at which growth is set by fixing glucose uptake, we plot Δ vs. $v_{\text{gro}}/v_{\text{gro}}^{\text{max}}$, where $v_{\text{gro}}^{\text{max}}$ is the maximum growth rate achieved fixing glucose uptake at its maximum physiological value (*v*_*Glc*_ = 10) and *v*_*gro*_ is the maximum growth rate obtained fixing glucose uptake at lower values (Figure 5, red squares). The curve obtained shows the same behavior as the upper curve of Figure S1 (Additional file 3), because glucose uptake limits *v*_*gro*_ and this, in turn, limits Δ.

A different way to constrain growth is fixing its upper bound ($v_{\text{gro}}^{\mathrm{ub}}$), its lower bound ($v_{\text{gro}}^{\mathrm{lb}}$) or both, without fixing the bounds of the external rates. In this way, we restrict the range of values that *v*_*gro*_ takes without altering the maximum potential growth that could be achieved. In the following curves, $v_{\text{gro}}^{\text{max}}$ is the maximum growth rate obtained in reference conditions. In green square curve of Figure 5, *v*_*gro*_ represents $v_{\text{gro}}^{\mathrm{ub}}$ ($v_{\text{gro}}^{\mathrm{lb}}$ being zero). In all the interval of $v_{\text{gro}}^{\mathrm{ub}}/v_{\text{gro}}^{\text{max}}$, Δ is approximately equal to one. Flux variability is insensitive to the value at which $v_{\text{gro}}^{\mathrm{ub}}$ is set. In black circle curve, *v*_*gro*_ represents $v_{\text{gro}}^{\mathrm{lb}}$ ($v_{\text{gro}}^{\mathrm{ub}}$ being $v_{\text{gro}}^{\text{max}}$) and, in blue circle curve, we fix both bounds at the same value ($v_{\text{gro}}=v_{\text{gro}}^{\mathrm{ub}}=v_{\text{gro}}^{\mathrm{lb}}$, [23]). These two curves show almost exactly the same behavior, Δ decreasing in approximately linear way with $v_{\text{gro}}/v_{\text{gro}}^{\text{max}}$. We conclude that to obtain high flux variability two necessary conditions must be fulfilled: i) the glucose uptake is such that a high growth rate can in principle be achieved (red square curve) and ii) the growth rate can take much lower values than the maximum growth rate (green square, black circle and blue circle curves). Black circle and blue circle curves are almost identical, indicating that fixing $v_{\text{gro}}^{\mathrm{lb}}$ alone or $v_{\text{gro}}^{\mathrm{lb}}$ and $v_{\text{gro}}^{\mathrm{ub}}$ has the same effect on variability. Therefore, the relevant source of flux variability is the value at which $v_{\text{gro}}^{\mathrm{lb}}$ is set; while the change in *v*_*gro*_, which is fixed in blue circle curve does not play a significant role. Substantial flux variability requires growing at a lower rate than the maximum growth that could be achieved for the external conditions considered. In this way, the resources that are not forced into biomass production can be allocated to other processes, increasing variability. The same results were obtained for anaerobic conditions (see Additional file 5). In what follows, we call the trade-off between growth rate and flux variability the growth-flexibility trade-off.

### Global reorganization during adaptations

**Figure 6:**
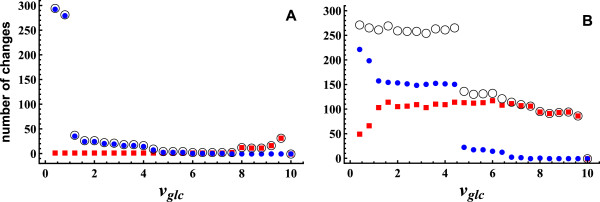
**Number of flux changes vs. glucose uptake.** Starting from the optimal growth state (glucose uptake 10), minimization of flux adjustment was used to calculate the final states (glucose uptakes smaller than 10). We show the number of fluxes that increase to more than double (red square curve), the number of fluxes that decrease to less than one tenth (blue circle curve) and their sum (black circle curve). In **A**, growth rate is fixed at the maximum value. High numbers of changing fluxes only appear for low glucose uptakes, when a substantial proportion of the fluxes are forced to take very low values. For this type of adaptations, large changes in only a small proportion of the fluxes take place. In **B**, growth rate is free. The number of fluxes that change is high in all the interval of glucose uptakes. Adaptations with growth variation produce global reorganization of the flux pattern in all the interval of glucose uptakes.

Adaptations to changes in environmental conditions may produce global reorganization of the metabolic network [11-16,24,25]. The question is if global reconfiguration of network fluxes depends on growth being a source of variation during adaptation. To test this hypothesis, we assume that *E. coli* adapts to changes in glucose uptake staying as close as possible to the initial state, i.e. minimizing flux adjustment. This principle, called Minimization of Metabolic Adjustments (MOMA, [26]), has been used to predict fluxes of central carbon metabolism [26] and lethality of deletion mutants [27]. Notably, it was recently shown that flux data obtained from *E. coli* is consistent with a scenario where flux states evolve under the trade-off between two principles: near optimality under one given condition and minimal adjustment between conditions [28]. Here, we assume a very similar type of scenario, i.e. optimal growth in the initial condition and minimization of flux adjustment determining the final condition. In the initial state glucose uptake is fixed at 10, the final states being defined by fixing glucose uptake at lower values. To assess the consequences of growth variation, the final state is calculated in two different ways: leaving growth free and fixing growth at the maximum value. When growth is free, we obtain a smaller distance because the three components of flux variability are used for flux adjustment while when growth is fixed only internal and external variability may be used (see Additional file 6). To measure the degree of reorganization during glucose uptake transitions we determine the number of fluxes that increase to more than double or decrease to less than one tenth, with respect to the initial state. The asymmetry of the interval chosen was introduced to take into account that the sum of the fluxes, in optimal growth conditions, is approximately proportional to glucose uptake (see Additional file 7), making more likely flux decreases than flux increases when glucose uptake is decreased. In Figure 6, we show the number of fluxes that increase to more than double, the number of fluxes that decrease to less than one tenth and their sum as a function of glucose uptake, fixing growth (Figure 6A) and leaving growth free (Figure 6B). When growth is fixed, the number of fluxes that change is small in almost all the interval of glucose uptake values. A high number of changes only appears when glucose uptake is very low, because the proportional decrease in the sum of the fluxes forces a substantial proportion of the fluxes to take very low values (Figure 6A). In contrast, when growth is free, the number of fluxes that change is high in all the interval of glucose uptakes (Figure 6B). These results show that under the principle of minimal flux adjustment, when adaptation occurs with growth variation, the network presents global reorganization of the flux pattern while, when adaptation occurs keeping growth at its maximum value, large changes in only a small proportion of the fluxes take place.

## Discussion

### Physiological regulation of flux variability

Our theoretical results show that the modulation of global flux variability requires that growth rate is regulated in response to changes in environmental conditions. Growth rate regulation has been an important topic in bacterial physiology for more than half a century. To date, many important aspects of the mechanisms involved have been elucidated [21]. A simplified description of the mechanism in *E. coli* is a follows. Changes in the nutrient richness or quality in the growth media regulate the activities of RelA (ppGpp synthetase) and SpoT (ppGpp synthetase and hydrolase), which in turn are responsible for any accumulation of ppGpp. Accumulation of ppGpp inversely correlates with nutrient richness or quality. It regulates rRNA synthesis and, as a consequence, growth rate. For instance, when fast growing cells are subject to amino acid starvation, inducing the stringent response [29], the ppGpp synthetase activity of RelA is activated, with a rapid accumulation of ppGpp. This is immediately followed by a shutoff of rRNA synthesis and growth arrest. *E. coli* reorganizes its global gene expression when faced with starvation [24,25]. ppGpp controls this massive response by coordinating the down-regulation of genes of the translation apparatus and the induction of the general stress response. In summary, starvation activates a cascade of signaling events that changes growth rate, producing a reconfiguration in the metabolic network. Note that the sequence of changes is not environment/growth/gene expression but environment/gene expression/growth. In this type of strategy, signaling pathways must be fine-tuned to set growth at the evolutionary adjusted rate for each specific environment, as also appears to be the case in the budding yeast [30]. The empirical mechanism by which *E. coli* regulates its growth rate is in agreement with our theoretical finding, suggesting that metabolic adaptations to environmental challenges require active mechanisms that reduce growth rate in order to increase the repertoire of responses. The fact that a global reorganization takes place during *E. coli* adaptation controlled by ppGpp is also consistent with growth variation being the source of flux variability, as was shown above (see Figure 6).

In a recent study [22], protein copy number distributions in *E. coli*[31] are used to impose constraints on fluxes in a metabolic reconstruction [20]. Distributions of 352 metabolic proteins are sampled to define the state of a unique cell in a population of 1 million. FBA [18] is used to determine the optimal growth solution for every cell. One central result of this study is that a large portion of the overall cell-to-cell variability in the flux patterns can be achieved by sampling a small number of enzymes most likely to constrain cell growth [22]. Our analysis, showing that growth variability is the only significant component of flux variability under physiological conditions, gives a straightforward interpretation to this finding.

Metabolic flux patterns of microorganisms in different environmental conditions may be best described by different optimality principles [32]. For instance, under certain conditions the optimality principle describing metabolic properties could be maximization of ATP yield. Calculations with the *iJO1366* model show that if the target is maximization of ATP yield, the maximum growth rate that could be achieved is reduced to zero. As a consequence, to keep ATP yield at its maximum value it is necessary to employ all growth variability, the three components of flux variability being negligible.

### Growth-flexibility trade-off and fitness

The fittest organism is, roughly speaking, the fastest growing organism that responds in the appropriate ways to environmental challenges. According to the growth-flexibility trade-off (Figure 5), growth rate is reduced during adaptation to an adverse environment, increasing metabolic flexibility to respond in the appropriate way to the new conditions. Thus, being able to achieve a high growth rate is advantageous in two respects. For constant environmental conditions, where fitness increases with growth rate, growth may be set at a high value. For fluctuating environments, the greater the potential growth is the greater is the metabolic flexibility and adaptation capacity which can be obtained reducing growth to suboptimal values. Therefore, maximizing the potential growth rate would contribute to increase fitness in both constant and fluctuating environments.

There is a strong parallelism between the growth-flexibility trade-off and the transition between the survival of the fittest and the survival of the flattest [33,34]. According to the fittest-flattest transition, for low mutation rates, the organism tends to display the maximum replication rate that could be achieved in those conditions. When mutation rate increases to high values, lower replication rates are favored, because these genotypes are located in flatter regions of the fitness surface and are, therefore, more robust with respect to mutations. Similarly, according to the growth-flexibility trade-off, in an approximately constant environment, the organism tends to display the maximum growth rate that could be achieved in those conditions. Under changing environmental conditions, lower growth rates are favored, because these phenotypes present higher volumes of alternative solutions and are, therefore, more robust with respect to external perturbations.

In addition to the parallelism between the growth-flexibility trade-off and the fittest-flattest transition, the effects of mutations and environment are not independent. There is evidence supporting the hypothesis that systems robust to environmental changes are also robust to mutations [35]. The essential idea is that physiological mechanisms that allow organisms to adjust to changing environments, such as by regulating gene expression, will also compensate for the effects of many mutations [36]. In the context of the growth-flexibility trade-off, the regulation of flux variability follows the sequence of changes environment/gene expression/growth. As a consequence, deleterious mutations, affecting gene expression in similar ways as harmful environmental changes, increase metabolic flexibility at the expense of optimal growth. The increase in the repertoire of alternative flux patterns obtained in this way makes more likely that the detrimental effects of mutations could be neutralized.

In general, our results show that the growth-flexibility trade-off would operate in all evolutionary adaptations taking place in environments with the conditions of the glucose minimal medium, with the only requirement that the stoichiometric network and the carbon-nitrogen coordination remain unchanged. An example is key innovations, where the evolution of a regulatory mechanism allows the expression of a catalytic activity in conditions where it was not previously expressed [37,38]. In contrast, evolved organisms presenting new catalytic activities are represented by expanded stoichiometry matrices. In this type of evolutionary adaptations, the existence of a growth-flexibility trade-off must be tested using the stoichiometry matrix corresponding to the evolved strain. Examples are pathogenic *E. coli* strains, showing different metabolites and reactions from the *iJO1366* genome-scale reconstruction (representing the *E. coli* K-12 MG1655 commensal strain) used in the present work. We tested the growth-flexibility trade-off in the *iEco1344_EDL933* genome-scale reconstruction [39], corresponding to *E. coli* O157:H7 EDL933 enterohemorrhagic strain. With this model of the pathogenic strain we arrive to the same general conclusions, namely, introducing the coordination of carbon and nitrogen utilization as an additional constraint, growth variability is the only significant component of flux variability in the physiological range of glucose, oxygen and ammonia uptakes (see results in Additional file 8). Further analysis of the growth-flexibility trade-off in other pathogenic *E. coli* strains [39,40] is under progress.

### Validity of the results obtained

The growth-flexibility trade-off obtained in this work only depends on the stoichiometry of the *iJO1366* reconstruction [19], the bounds of particular fluxes and the coordination of carbon and nitrogen uptakes [20]. The treatment does not include additional constraints imposed by rate equations, parameter values or allosteric interactions. If these were considered, our results showing that internal and external variability make no significant contribution to metabolic flexibility would remain valid, because a description where rates are further constrained may not show higher internal and external variability.

A valid question would be how the results obtained depend on the measure of flux variability used. In this work we used Δ, representing the average variability of individual fluxes. An alternative way to quantify flux variability would be to calculate the variability of the average flux (Σ). This is defined as the difference between the maximum and minimum values that the average flux can take, divided by the same quantity evaluated at the reference conditions: $\Sigma =\left({\left(\sum_{i=1}^{r}j_{i}\right)}_{\text{max}}-\;{\left(\sum_{i=1}^{r}j_{i}\right)}_{\text{min}}\right)/\left({\left(\sum_{i=1}^{r}V_{i}\right)}_{\text{max}}-\;{\left(\sum_{i=1}^{r}V_{i}\right)}_{\text{min}}\right)$. The components of flux variability, that we previously defined in terms of Δ (Δ_*int*_, Δ_*ext*_ and Δ_*gro*_), may be determined using Σ (Σ_*int*_, Σ_*ext*_ and Σ_*gro*_, with Σ_*tot*_ = Σ_*int*_ + Σ_*ext*_ + Σ_*gro*_). Note that the calculation of Σ, for each new condition, requires solving 2 LP problems, which is much faster than the calculation of Δ. The analysis described in section *Flux variability vs. uptake rates* was performed using the alternative measure of flux variability, Σ, arriving to the same general conclusions (see Additional file 9).

The measure of flux variability used quantifies the global behavior of the network, only changes in a large proportion of the rates producing significant effects. It is, therefore, likely that the general conclusions obtained with the *iJO1366* model [19] are robust to missing reactions or errors in the reconstruction. To test this hypothesis, we repeated the calculations with a previous reconstruction, published a decade ago (*iJR904*, [41]), including less than 1000 metabolic reactions (San Román M (2013) MSc. Thesis, Universidad de la República, Montevideo, Uruguay), and compared the results obtained with the latest reconstruction (*iJO1366*, [19]), which accounts for more than 2200 reactions. Notably, with the two models we arrive to the same general conclusion: introducing the coordination of carbon and nitrogen utilization as an additional constraint, growth variability is the only significant component of flux variability in the physiological range of glucose, oxygen and ammonia uptakes (calculation of flux variability in *iJR904* model and the results obtained are described in Additional file 10). Finally, we repeated the calculations with a network reconstruction of intermediate size, *iAF1260*[42]. The results obtained also showed the same behavior as *iJO1366*[19], as expected. All these results suggest that the conclusions of this work are highly robust to missing reactions or errors in the reconstruction.

### Final comments and perspective

The results presented in this work show that, for *E. coli* growing in the conditions of glucose minimal medium, the growth-flexibility trade-off is the only significant source of metabolic flexibility. Internal and external variability are shown not to be significant components of variability of the internal fluxes. The question is why this is the case. For *E. coli* in the wild, external rates are affected by unpredictable environmental changes, external variation appearing to be unsuitable to modulate metabolic flexibility. On the other hand, it could be argued that external variation could be modulated by the same physiological mechanisms which are used to adjust uptake rates in response to external changes. However, as we saw, one of these fundamental mechanisms, coordinating carbon and nitrogen uptake [20], has precisely the opposite effect, i.e. eliminating external (and internal) variability. Regarding internal variation, it is very difficult to conceive a mechanism where the global variability of the internal reactions is efficiently regulated by the intrinsic variation of the same reactions. Growth variation is the remaining source of metabolic flexibility. The analysis presented in this work points to growth rate regulation as the mechanism modulating metabolic flexibility in *E. coli* growing on glucose. Work is in progress to determine if the same principle operates in other environmental conditions and organisms.

## Conclusions

A procedure is devised to classify and quantify the sources of flux variability. To our knowledge, this is the first method proposed for this purpose. The variability of the internal reactions may be decomposed into three components: internal, external and growth variability. Growth variability is the only significant component of flux variability in all physiological conditions studied. The increase in metabolic flexibility requires reducing growth to suboptimal values. This growth-flexibility trade-off is the only way that *E. coli* has to change its global flux variability. We show that the puzzling phenomenon of global reorganization occurring during adaptations to environmental challenges [11-16,24,25] may be obtained as a consequence of the growth-flexibility trade-off. These conclusions only depend on the constraints of the *iJO1366* genome-scale model [19] and the coordination of carbon and nitrogen utilization, recently described in *E. coli*[20]. Their validity does not depend on the kinetic constraints imposed by the rate laws of the reactions. In addition, the conclusions are highly robust to incomplete or incorrect knowledge of the reaction network.

## Methods

### Flux Balance Analysis (FBA)

In FBA, LP is used to find a steady-state flux distribution (*v*) that maximizes growth rate (*v*_*gro*_) under mass balance, thermodynamical and flux capacity constraints. The LP problem can be formalized as follows [18]:

$$\text{max}\;v_{\text{gro}}\;$$

s.t.Nv=o

$$\alpha_{i}\leq v_{i}\leq \beta_{i},i\in R$$

Mass balance constraints are imposed by a system of linear equations where *N* is an *mxr* stoichiometry matrix, where *M* = {1, …, *m*} is the set of metabolite indexes and *R* = {1, …, *r*} is the set of reaction indexes. Thermodynamic constraints that restrict flow direction and capacity constraints are imposed by setting *α* and *β* as lower and upper bounds on flux values.

### Minimization of Metabolic Adjustments (MoMA)

Linear MoMA finds a solution that satisfies the same constraints as FBA while minimizing the Manhattan distance from a reference flux distribution. Linear MoMA is formalized as follows [26]:

$$\text{min}\sum_{i=1}^{r}\left|v_{i}-v_{i}^{\mathrm{ref}}\right|$$

s.t.Nv=o

$$\alpha_{i}\leq v_{i}\leq \beta_{i},i\in R$$

In this work, $v_{i}^{\mathrm{ref}}$ is the flux distribution found with FBA setting glucose uptake at 10.

### Flux Variability Analysis (FVA)

FVA is used to quantify the feasible solution space. For this, the maximum and minimum flux value of each reaction *i*, under the same constraints as in FBA, is found. The LP problem can be formalized as follows [17]:

$$\text{max}\left(\text{min}\right)\;v_{i}$$

s.t.Nv=o

$$\alpha_{i}\leq v_{i}\leq \beta_{i},i\in R$$

Flux variability is quantified relative to a reference state [23]:

$$\Delta =\frac{1}{r}\;\sum_{i=1}^{r}\frac{j_{i}^{\text{max}}-\;j_{i}^{\text{min}}}{V_{i}^{\text{max}}-\;V_{i}^{\text{min}}}$$

where $V_{i}^{\text{max}}$ and $V_{i}^{\text{min}}$ are the maximum and minimum flux values of reaction *i* in the reference conditions, and $j_{i}^{\text{max}}$ and $j_{i}^{\text{min}}$ are the same values under other conditions. The sum is over the *internal* reactions. The rates are classified in three groups: growth rate (*v*_*gro*_), external rates (*v*_*ext*_) and internal rates (*v*_*int*_). We call $v_{\text{gro}}^{\text{max}}$ to the maximum growth rate and $v_{\text{ext}}^{\text{max}}$ to the values of the external rates for the optimal growth solution. Δ is calculated imposing three different sets of constraints on the rates: a) $v_{\text{gro}}=v_{\text{gro}}^{\text{max}}$, $v_{\text{ext}}=v_{\text{ext}}^{\text{max}}$ and *v*_*int*_ free (Δ_*int*_), b) $v_{\text{gro}}=v_{\text{gro}}^{\text{max}}$, *v*_*ext*_ free and *v*_*int*_ free (Δ_*int* + *ext*_), and c) *v*_*gro*_, *v*_*ext*_ and *v*_*int*_ all free (Δ_*tot*_). Finally, Δ_*ext*_ = Δ_*int* + *ext*_ − Δ_*int*_ and Δ_*gro*_ = Δ_*tot*_ − Δ_*int* + *ext*_.

FBA, FVA and linear MoMA are formulations of linear programming (LP). The open source GNU Linear Programming Kit (GLPK) (http://www.gnu.org/software/glpk) was used for solving all the LP problems included in this work.

## Competing interests

The authors declare that they have no competing interests.

## Authors’ contributions

MSR participated in the design of the study, performed the calculations and participated in the analysis of the results. HC participated in the design of the study and participated in the analysis of the results. LA participated in the design of the study, participated in the analysis of the results and drafted the manuscript. All authors read and approved the final manuscript.

## Supplementary Material

Additional file 1 **Genome scale *in silico *** **models and flux variability analysis (additional considerations).** Click here for file

Additional file 2 **Glucose minimal medium for *iJO1366 in silico *** **model.** Click here for file

Additional file 3 Growth rate vs. glucose uptake. Click here for file

Additional file 4 Flux variability vs. glucose uptake, in anaerobic conditions. Click here for file

Additional file 5 Flux variability vs. growth rate, in anaerobic conditions. Click here for file

Additional file 6 Flux adjustment vs. glucose uptake. Click here for file

Additional file 7 Sum of fluxes vs. glucose uptake. Click here for file

Additional file 8 **FVA in *iEco1344_EDL933 *** **model.** Click here for file

Additional file 9 Variability of the average flux vs. uptake rate. Click here for file

Additional file 10 **FVA in *iJR904 *** **model.** Click here for file
