# Supplementary material for: Source and regulation of flux variability in Escherichia coli
Source: BMC Syst Biol. 2014 Jun 14;8:67. doi: 10.1186/1752-0509-8-67 (PMC4074586; doi:10.1186/1752-0509-8-67)
Supplement: Additional file 1 — Genome scale in silico models and flux variability analysis (additional considerations). [file 1752-0509-8-67-S1.pdf]

Additional file 1: Genome scale *in silico* models and flux variability analysis (additional considerations).

1. Genome-scale *in silico* models

The metabolic maps for particular strains of microorganisms may be reconstructed from genomic, biochemical, physiological and genetic information, among others. These maps render the stoichiometric coefficients of all the biochemical reactions that can be taking place in any condition. For a system with m metabolites and r reactions, the stoichiometric coefficients are arranged in a stoichiometry matrix, N, having m rows and r columns. The element N_{ik} is the stoichiometric coefficient of the i th metabolite taking part in the k th reaction. In this work, we use the *E. coli* stoichiometry matrices of the *iJO1366* ($m = 1136$ and $r = 2251$) (1) and *iJR904* ($m = 904$ and $r = 1075$) (2) *in silico* models. The rates of the reactions are arranged in a column vector, v . At steady state, the rates fulfill the matrix equation: $N\, v = o$, where o is the null vector. The rates are functions of the internal metabolite concentrations and parameters. If these kinetic laws were replaced in the matrix equation, the metabolite concentrations and fluxes could be calculated (numerically) as a function of the parameters. However, we still lack reliable information on the kinetic laws and parameter values governing the rates of the metabolic processes *in vivo*, hindering the possibility to perform, with confidence, this type of analysis. On the other hand, we can consider the rates as the dependent variables, and solve $N\, v = o$ for these. This type of approach, which does not rely on detailed kinetic information, is the one used in the present work (3).

Reactions of metabolic networks are of three types: external, growth and internal. External reactions are auxiliary rates that produce or consume the metabolites external to the cell, maintaining their concentrations constant. They are introduced to ensure the steady state of the system. There is one external rate for each external metabolite. Growth reactions consume metabolic intermediates to produce biomass. Finally, internal reactions are all the remaining reactions, including the membrane transport processes and the metabolic machinery. There are limits to the range of values that the rates of the reactions can take. The upper and lower bounds depend on physical constraints or conditions imposed by the particular properties of the medium. For example, irreversible reactions are either positive or negative, being one of their bounds zero, and the upper bound of rates of nutrient incorporation to the cell cannot exceed the maximum transport rate through the plasma membrane.

In all well defined genome-scale metabolic reconstructions, there are more reactions (i.e. columns of N) than independent metabolites (i.e. linearly independent rows of N), the system being underdetermined and showing an infinite number of solutions. Selecting particular flux distributions, from the set of infinite solutions, requires imposing additional criteria. One possibility is the optimization of a given objective function (which can be any variable or linear combination of variables of the system), for example, maximizing the growth rate, as in flux balance analysis (FBA) (3). However, even after the additional criteria are imposed, there may still be more than one alternative solution, satisfying all the constraints.

2. FVA in genome-scale metabolic reconstructions

One simple approach to quantify changes in the high dimensional volume of alternative solutions is using Flux Variability Analysis (FVA) (4). The absolute flux variability of reaction i, in given conditions, may be calculated as the difference $j_i^{max} - j_i^{min}$, j_i^{max} and j_i^{min} being the maximum and minimum flux values of reaction i, respectively. The differences $j_i^{max} - j_i^{min}$ correspond to the edges of the smallest hyper-rectangle containing the volume of alternative

solutions. The variability of reaction i, in certain conditions, may be expressed relative to the variability in reference conditions: $\delta_i = \left(j_i^{max} - j_i^{min}\right) / \left(V_i^{max} - V_i^{min}\right)$, V_i^{max} and V_i^{min} being the maximum and minimum flux values of reaction i in the reference conditions. The average flux variability is: $\Delta = \sum_{i=1}^{r} \delta_i \Big/ r$ (4, 5), where the sum is over the *internal* reactions.

Reactions where minimum and maximum flux values, as computed by flux variability analysis (FVA) (4), coincide do not contribute to flux variability. These uniquely determined reactions are of two types: fixed and blocked. In the case of fixed reactions, the maximum and minimum flux values coincide and are different from zero. In the *iJR904* model there is one fixed reaction in all conditions: the flux of ATP consumption for cell maintenance ($v_{ATPm} = 7.6$). The *iJO1366* model has no fixed reactions. Note that other fluxes are fixed for particular conditions, as it is the case for oxygen uptake when the models are studied in anaerobic conditions, in which case oxygen uptake is set to zero. Blocked reactions, on the other hand, are those reactions with zero flux, under given conditions (6). In *iJO1366* there are 878 blocked reactions in glucose minimal medium, 213 of which are external, and in *iJR904*, there are 408 blocked reactions in glucose minimal medium, 86 of which are external. Allowing a maximum glucose uptake of 20 (instead of 10) does not change the number of reactions that are fixed or blocked neither in *iJO1366* nor in *iJR904*. Fixed and blocked reactions found in glucose minimal medium were identified and not used in variability calculations because they do not contribute to flux variability. After excluding these, there are 1373 remaining reactions: 111 external, 2 growth ("wild type" and "core") and 1260 internal in *iJO1366* and there are 667 remaining reactions: 86 external, 1 growth and 580 internal in *iJR904*. The calculations with *iJO1366* are performed with the "core" biomass reaction (as in (1)). In addition to the calculation of the reference state, the calculation of the average flux variability (Δ) for each new condition requires solving 2520 LP problems in *iJO1366* and 1160 LP problems in *iJR904*. In *iJO1366* and *iJR904* some reactions show unbounded fluxes even if the nutrient uptake rates are bounded. The presence of these unbounded fluxes would violate the second law of thermodynamics, so that they should be eliminated from the model. We used the algorithm of Wright and Wagner (7) to identify these cycles and eliminate them from the variability calculations (see also (8)).

In this work, we do not include the external rates and the growth rate in the calculation of the average flux variability ($\Delta = \sum_{i=1}^{r} \delta_i \Big/ r$, where r is the number of *internal* reactions). External rates are auxiliary rates introduced for convenience to perform the calculations, their variability having no biological significance. The growth rate is a reaction introduced in the model to account for the complex sub-network of metabolic reactions consuming intermediates in the proportions of the cellular composition to produce biomass (3). Introducing this reaction in the calculation of Δ, which has a large number of terms, will produce no significant difference, potentially underestimating the contribution that the sub-network associated to growth could make to the average flux variability if it were displayed in all its extent.

References

[1] Orth JD, Conrad TM, Na J, Lerman JA, Nam H, Feist AM, Palsson BØ: **A comprehensive genome-scale reconstruction of *Escherichia coli* metabolism-2011.** *Mol Syst Biol* 2011, **7**:535.

[2] Reed JL, Vo TD, Schilling CH, Palsson BØ: **An expanded genome-scale model of *Escherichia coli* K-12 (iJR904 GSM/GPR).** *Genome Biology* 2003, **4**:R54.
[3] Orth JD, Thiele I, Palsson BØ: **What is flux balance analysis?** *Nat Biotechnol* 2010, **28**:245–248.
[4] Mahadevan R, Schilling CH: **The effects of alternate optimal solutions in constraint-based genome-scale metabolic models.** *Metab Eng* 2003, **5**:264–276.
[5] Chen T, Xie ZW, Ouyang Q: **Expanded flux variability analysis on metabolic network of *Escherichia coli*.** *Chinese Sci Bull* 2009, **54**:2610-2619.
[6] Burgard AP, Nikolaev EV, Schilling CH, Maranas CD: **Flux coupling analysis of genome-scale metabolic network reconstructions.** *Genome Res* 2004, **14**:301-312.
[7] Wright J, Wagner A: **Exhaustive identification of steady state cycles in large stoichiometric networks.** *BMC Systems Biology* 2008, **2**:61.
[8] Muller AC, Bockmayr A: **Fast thermodynamically constrained flux variability analysis.** *Bioinformatics* 2013, **29**:903-909.
