# Supplementary material for: Source and regulation of flux variability in Escherichia coli
Source: BMC Syst Biol. 2014 Jun 14;8:67. doi: 10.1186/1752-0509-8-67 (PMC4074586; doi:10.1186/1752-0509-8-67)
Supplement: Additional file 2 — Glucose minimal medium for iJO1366 in silico model. [file 1752-0509-8-67-S2.pdf]

Additional file 2: Glucose minimal medium for *iJO1366 in silico* model.

Table S1. **Reference conditions: Glucose minimal medium (*iJO1366* model)**[1]. The external input rates are defined as positive and the external output rates as negative.

External species (reaction)	Lower bound	Upper bound
Glucose (glc)	-1000	10
Oxygen (o2)	-1000	1000
Ammonium (nh4)	-1000	1000
Phosphate (pi)	-1000	1000
Sulfate (so4)	-1000	1000
Carbon dioxide (co2)	-1000	1000
Water (h20)	-1000	1000
Proton (h)	-1000	1000
Calcium (ca2)	-1000	1000
Cobalt (cobalt2)	-1000	1000
Cupper (cu2)	-1000	1000
Iron II (fe2)	-1000	1000
Iron III(fe3)	-1000	1000
Potassium (k)	-1000	1000
Magnesium (mg2)	-1000	1000
Manganese (mn2)	-1000	1000
Molybdenum (mobd)	-1000	1000
Nickel (ni2)	-1000	1000
Zinc (zn2)	-1000	1000
Chloride (cl)	-1000	1000

[1] fermentation products, not present in the medium, have upper bounds equal to zero.
