# Supplementary material for: Source and regulation of flux variability in Escherichia coli
Source: BMC Syst Biol. 2014 Jun 14;8:67. doi: 10.1186/1752-0509-8-67 (PMC4074586; doi:10.1186/1752-0509-8-67)
Supplement: Additional file 3 — Growth rate vs. glucose uptake. [file 1752-0509-8-67-S3.pdf]

Additional file 3: Growth rate vs. glucose uptake.

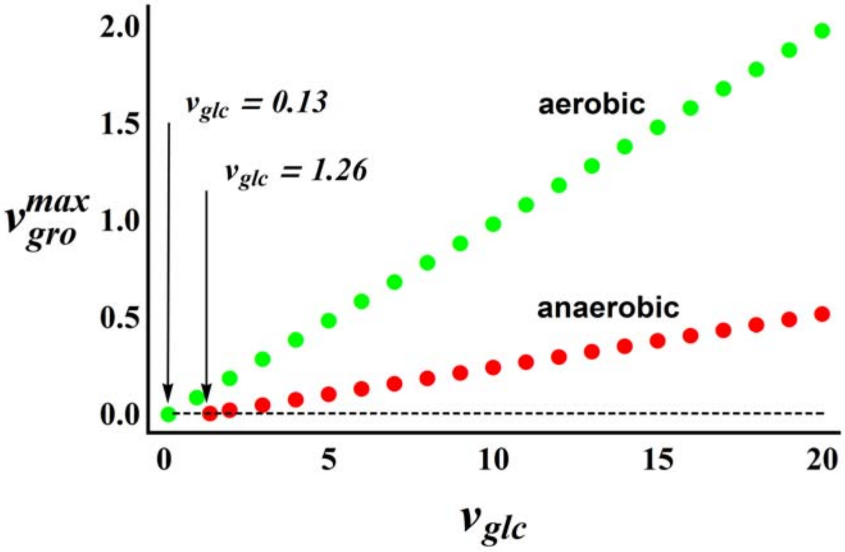

Figure S1. **Maximum growth rate vs. glucose uptake.** Aerobic (●) and anaerobic (●) conditions are represented. The minimum glucose uptakes to fulfill the requirements of ATP for cell maintenance are 0.13 and 1.26 for aerobic and anaerobic conditions, respectively.
