# Supplementary material for: Source and regulation of flux variability in Escherichia coli
Source: BMC Syst Biol. 2014 Jun 14;8:67. doi: 10.1186/1752-0509-8-67 (PMC4074586; doi:10.1186/1752-0509-8-67)
Supplement: Additional file 4 — Flux variability vs. glucose uptake, in anaerobic conditions. [file 1752-0509-8-67-S4.pdf]

Additional file 4: Flux variability vs. glucose uptake, in anaerobic conditions.

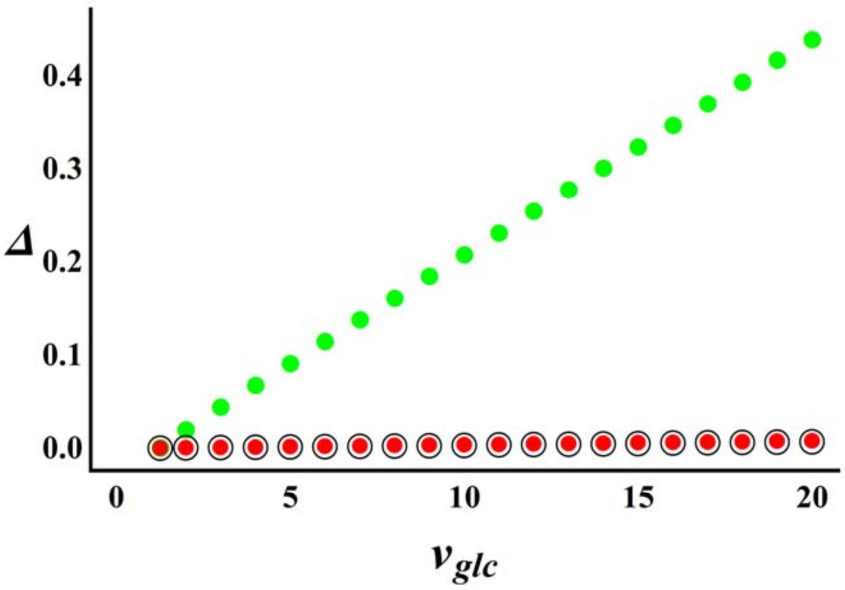

Figure S2. **Components of flux variability vs. glucose uptake (anaerobic conditions).** ○ represents Δ_{int}, ● represents $\Delta_{int} + \Delta_{ext}$ and ● represents $\Delta_{tot} = \Delta_{int} + \Delta_{ext} + \Delta_{gro}$. Δ_{gro} is the only significant component of flux variability, Δ_{int} and Δ_{ext} having negligible values for all the range of glucose uptakes studied (the physiological values of glucose uptake are between 0 and 10).
