# Supplementary material for: Source and regulation of flux variability in Escherichia coli
Source: BMC Syst Biol. 2014 Jun 14;8:67. doi: 10.1186/1752-0509-8-67 (PMC4074586; doi:10.1186/1752-0509-8-67)
Supplement: Additional file 5 — Flux variability vs. growth rate, in anaerobic conditions. [file 1752-0509-8-67-S5.pdf]

Additional file 5: Flux variability vs. growth rate, in anaerobic conditions.

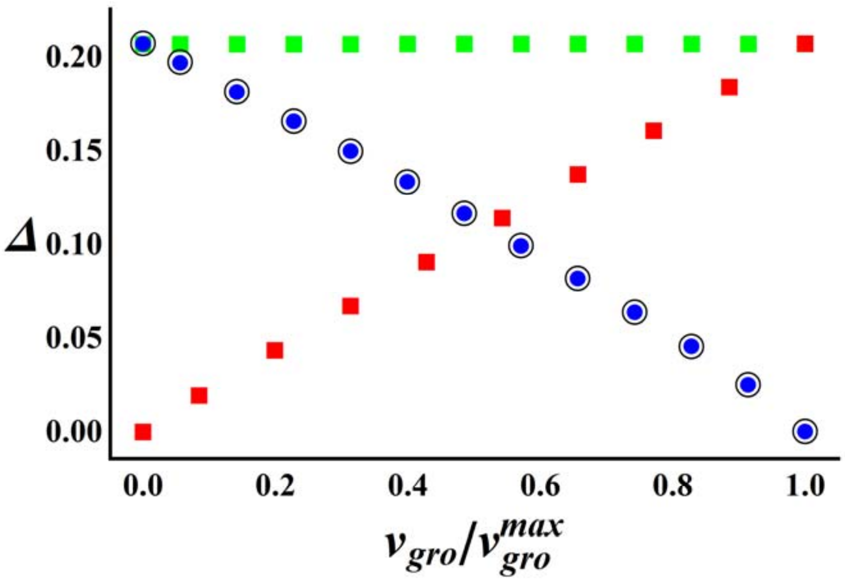

Figure S3. **Total flux variability vs. growth rate (anaerobic conditions).** ■ corresponds to fixing glucose uptake at different values between 0 and 10 (keeping growth free), ■ corresponds to fixing growth upper bound (keeping growth lower bound at zero and glucose uptake free), ○ corresponds to fixing growth lower bound (keeping growth upper bound at maximum growth and glucose uptake free) and ● corresponds to fixing upper and lower growth bounds at the same value (keeping glucose uptake free). Curve ■ shows that growth limitation by glucose uptake produces an approximately proportional reduction in flux variability. Curve ■ indicates that fixing growth upper bound has a negligible effect on flux variability. Curves ○ and ● show that fixing growth lower bound or both bounds have the same effect. Therefore, high flux variability requires that glucose uptake is high, so that the organism can grow at a potentially high rate, and that growth rate can be regulated to much lower values than the potential maximum growth.
