# Supplementary material for: Source and regulation of flux variability in Escherichia coli
Source: BMC Syst Biol. 2014 Jun 14;8:67. doi: 10.1186/1752-0509-8-67 (PMC4074586; doi:10.1186/1752-0509-8-67)
Supplement: Additional file 6 — Flux adjustment vs. glucose uptake. [file 1752-0509-8-67-S6.pdf]

Additional file 6: Flux adjustment vs. glucose uptake.

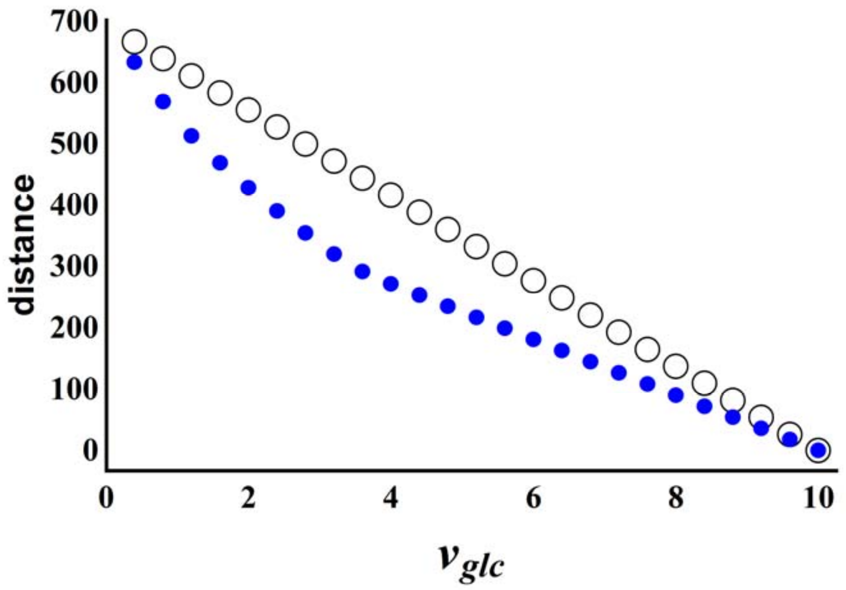

Figure S4. **Distance of flux adjustment vs. glucose uptake.** Starting from the optimal growth state (glucose uptake 10), minimization of flux adjustment was used to calculate the final states (glucose uptakes smaller than 10).We show the distance between the initial and final states fixing growth rate at the maximum value (curve ○) and leaving growth rate free (curve ●). Growth variation allows a better adjustment than when growth is fixed at its maximum value.
