# Supplementary material for: Source and regulation of flux variability in Escherichia coli
Source: BMC Syst Biol. 2014 Jun 14;8:67. doi: 10.1186/1752-0509-8-67 (PMC4074586; doi:10.1186/1752-0509-8-67)
Supplement: Additional file 7 — Sum of fluxes vs. glucose uptake. [file 1752-0509-8-67-S7.pdf]

Additional file 7: Sum of fluxes vs. glucose uptake.

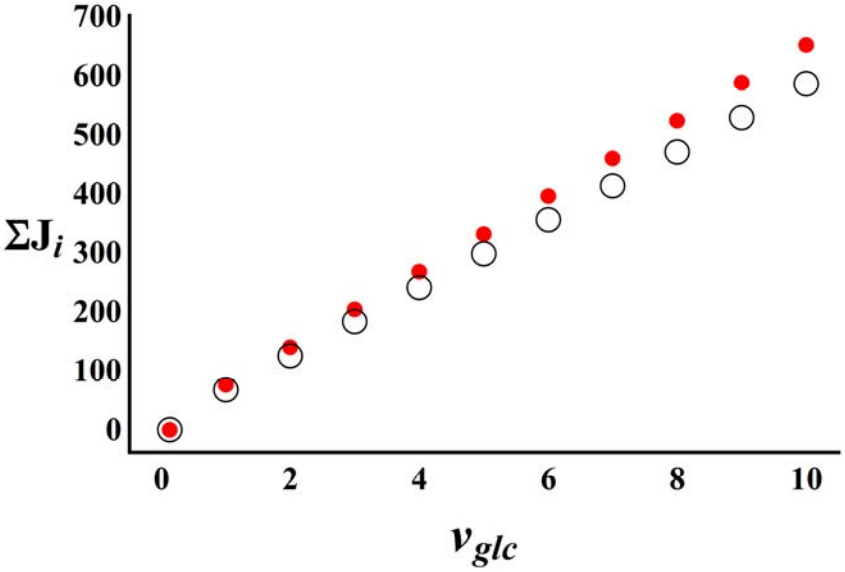

Figure S5. **Sum of fluxes vs. glucose uptake.** The sum of the fluxes was minimized (○) and maximized (●) in optimal growth conditions for fixed values of glucose uptake. The corresponding minimum and maximum sum of fluxes show very similar values. The sum of fluxes takes values approximately proportional to glucose uptake.
