# Supplementary material for: Source and regulation of flux variability in Escherichia coli
Source: BMC Syst Biol. 2014 Jun 14;8:67. doi: 10.1186/1752-0509-8-67 (PMC4074586; doi:10.1186/1752-0509-8-67)
Supplement: Additional file 8 — FVA in iEco1344_EDL933 model. [file 1752-0509-8-67-S8.pdf]

Additional file 8: FVA in *iEco1344_EDL933* model.

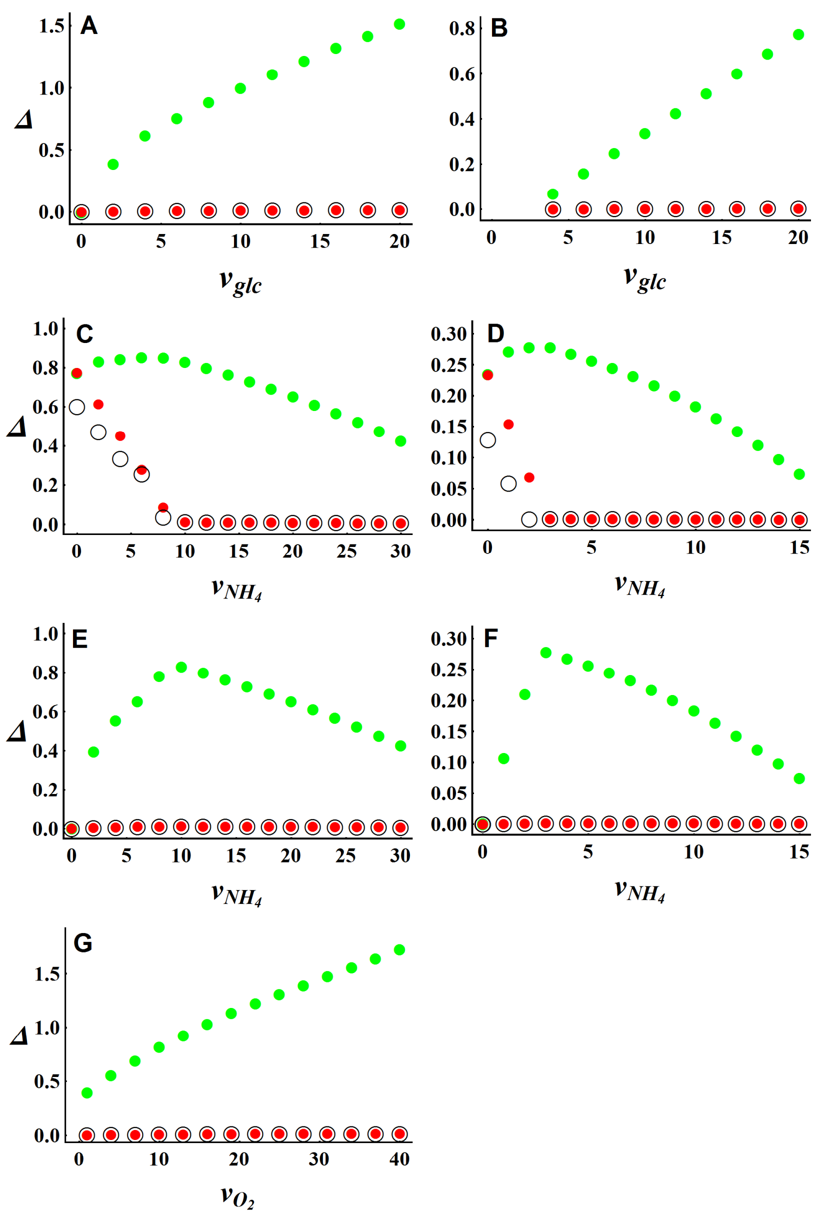

Figure S6. **Flux variability in *iEco1344_EDL933* model.** A, B, C, D, E, F and G, calculated for the *iEco1344_EDL933* model, correspond to Figs. 2, S2, 4A, 4B, 4C, 4D and 3, calculated for the *iJO1366* model, respectively. For the *iEco1344_EDL933* model, the only significant component of flux variability, in the physiological conditions studied, is growth variability.
