# Supplementary material for: Source and regulation of flux variability in Escherichia coli
Source: BMC Syst Biol. 2014 Jun 14;8:67. doi: 10.1186/1752-0509-8-67 (PMC4074586; doi:10.1186/1752-0509-8-67)
Supplement: Additional file 9 — Variability of the average flux vs. uptake rate. [file 1752-0509-8-67-S9.pdf]

Additional file 9: Variability of the average flux vs. uptake rate.

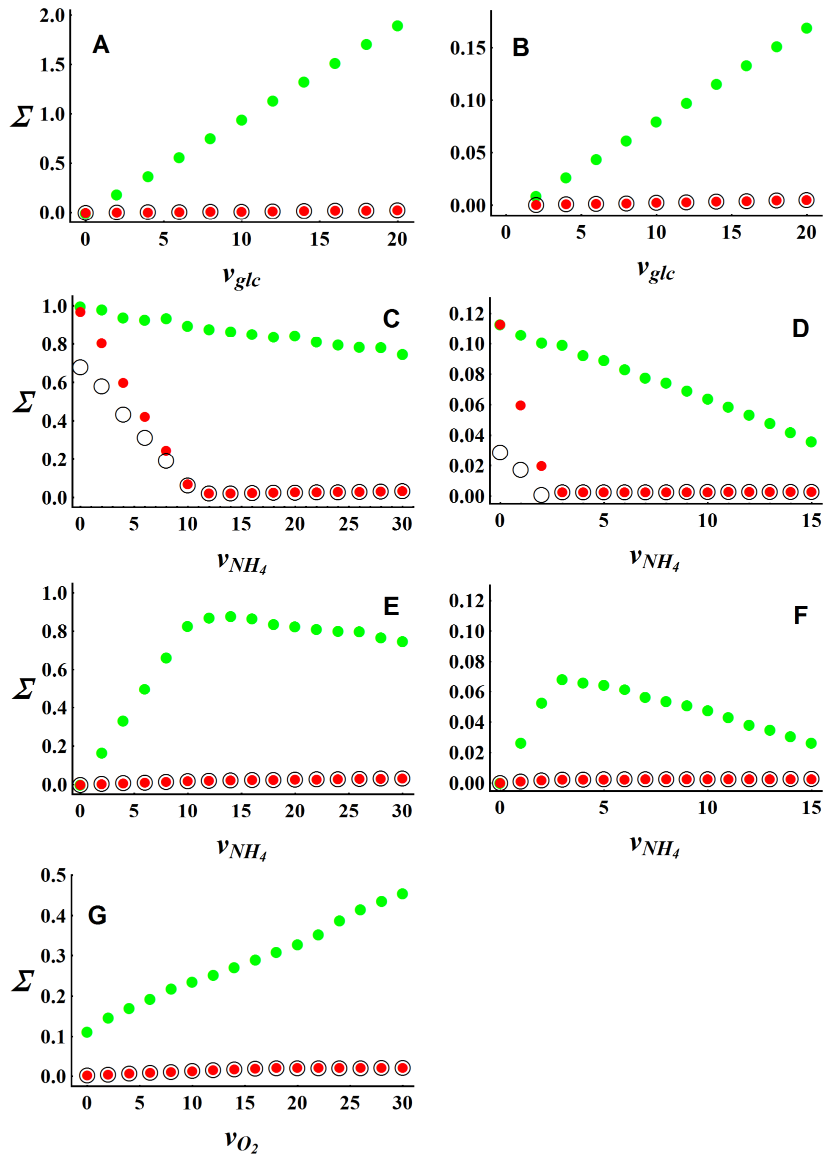

Figure S7. **Variability of the average flux vs. uptake rate.** A, B, C, D, E, F and G, calculated using Σ, correspond to Figs. 2, S2, 4A, 4B, 4C, 4D and 3 calculated using Δ, respectively. The results obtained with both measures of flux variability lead to the same general conclusions.
