# Supplementary material for: Source and regulation of flux variability in Escherichia coli
Source: BMC Syst Biol. 2014 Jun 14;8:67. doi: 10.1186/1752-0509-8-67 (PMC4074586; doi:10.1186/1752-0509-8-67)
Supplement: Additional file 10 — FVA in iJR904 model. [file 1752-0509-8-67-S10.pdf]

Additional file 10: FVA in *iJR904* model.

Flux variability analysis in the *iJR904 in silico* model

The reference conditions used to calculate Δ in the *iJR904* model (1) are those of the glucose minimal medium, described in Table S2 (see below). Note that an important difference with the *iJO1366* model (2) is that in the *iJR904* the physiological range of oxygen uptakes has an upper bound of 20. Another difference between the models is that, in the *iJR904* model, fixing phosphate or sulfate uptake blocks all potential variability increases by other external reactions (and their own variability, alone, cannot contribute to increase flux variability). This does not happen for the *iJO1366*. Therefore, in the calculations with *iJR904* we leave phosphate and sulfate uptakes free to move. In this way, the lack of internal or external variation is not due to this limiting property shown by *iJR904* (San Román M (2013) MSc. Thesis, Universidad de la República, Montevideo, Uruguay).

Δ_{tot} for the glucose minimal medium in aerobic conditions (Table S2) is, by definition, equal to 1. Imposing $v_{O_2} = 0$ we obtain: $\Delta_{tot} = 0.266$, which is the maximum variability that can be achieved for the glucose minimal medium in anaerobic conditions. Thus, the absence of oxygen reduces the variability to approximately one fourth of the reference value.

Figures S7A, S7B, S7C, S7D, S7E, S7F and S7G, calculated for the *iJR904* model (see below), correspond to Figures 2, S2, 4A, 4B, 4C, 4D and 3, calculated for the *iJO1366* model, respectively. From these figures, we can conclude that, also for the much smaller *iJR904* model, the only significant component of flux variability is growth variability, in the physiological conditions studied. One important difference appears in Figure S7G, because in the *iJR904* model the contribution of internal and external variability starts at oxygen uptake equals 20 (the boundary between physiological and non-physiological oxygen uptakes in the *iJR904* model, Table S2). This difference could be eliminated by introducing in the incomplete *iJR904* model the phenomenon called overflow metabolism, as is described next.

Overflow metabolism in *Escherichia coli* consists on acetate excretion at high rates of glucose uptakes, even in the presence of abundant oxygen (3). Acetate is produced when acetyl-coenzyme A is directed to acetate instead of entering the tricarboxylic acid cycle. Acetate excretion starts at a glucose uptake rate of, approximately, $5\, mmol/gDW/h$. Transcription studies show that several genes involved in the tricarboxylic acid cycle and respiration are repressed as the glucose uptake rate is increased. These and other experiments point to an explanation of overflow metabolism by a transcriptional limitation in the respiratory capacity (3).

In the *iJR904* model under reference conditions (Table S2), the maximum glucose and oxygen uptakes are 10 and 20, respectively. In our previous calculations, maximization of growth consumes the maximum glucose and oxygen uptakes, all the glucose being transformed into CO_2 by respiration. This behavior of the *iJR904* is not in agreement with the experimental results described above (acetate excretion starting at, approximately, $v_{Glc} = 5$). To introduce in the model the physiological constraints associated with overflow metabolism in a simple (though rough) way, we assume that at $v_{Glc} = 5$ respiration is working at its maximum capacity. This is imposed by setting the upper bounds of the rates of cytochromes *bd* and *bo3* to the values obtained for $v_{Glc} = 5$ (i.e. $v_{bd}^{ub} = 0$ and $v_{bo3}^{ub} = 21.45$). For higher glucose uptakes, no additional oxygen is consumed in the respiratory chain, additional inputs of glucose being transformed into fermentation products. In Figure S7H, we plot Δ vs. oxygen uptake, under the constraints associated with overflow metabolism ($v_{bd}^{ub} = 0$ and

$v_{bo3}^{ub} = 21.45$). In the range of oxygen uptakes studied, Δ_{gro} is the only significant component of flux variability. The relevant difference with the calculations performed in the absence of overflow metabolism (Figures S7G) is that, for oxygen uptakes between 20 and 40 (defined as non-physiological in *iJR904*), Δ_{int} and Δ_{ext} make no significant contribution to flux variability.

References

[1] Orth JD, Thiele I, Palsson BØ: **What is flux balance analysis?** *Nat Biotechnol* 2010, **28**:245–248.
[2] Orth JD, Conrad TM, Na J, Lerman JA, Nam H, Feist AM, Palsson BØ: **A comprehensive genome-scale reconstruction of *Escherichia coli* metabolism-2011.** *Mol Syst Biol* 2011, **7**:535.
[3] Vemuri GN, Altman E, Sangurdekar DP, Khodursky AB, Eiteman MA: **Overflow metabolism in *Escherichia coli* during steady-state growth: Transcriptional regulation and effect of the redox ratio.** *Appl Environ Microbiol* 2006, 72:3653-3661.

Table S2. **Reference conditions: Glucose minimal medium (*iJR904 in silico* model)**[1]. The external input rates are defined as positive and the external output rates as negative.

External reaction (name)	Lower bound	Upper bound
Glucose (glc)	0	10
Oxygen (o2)	0	20
Ammonium (nh4)	-1000	1000
Phosphate (pi)	-1000	1000
Sulfate (so4)	-1000	1000
Carbon dioxide (co2)	-1000	1000
Water (h20)	-1000	1000
Proton (h)	-1000	1000

[1] fermentation products, not present in the medium, have lower bounds equal zero

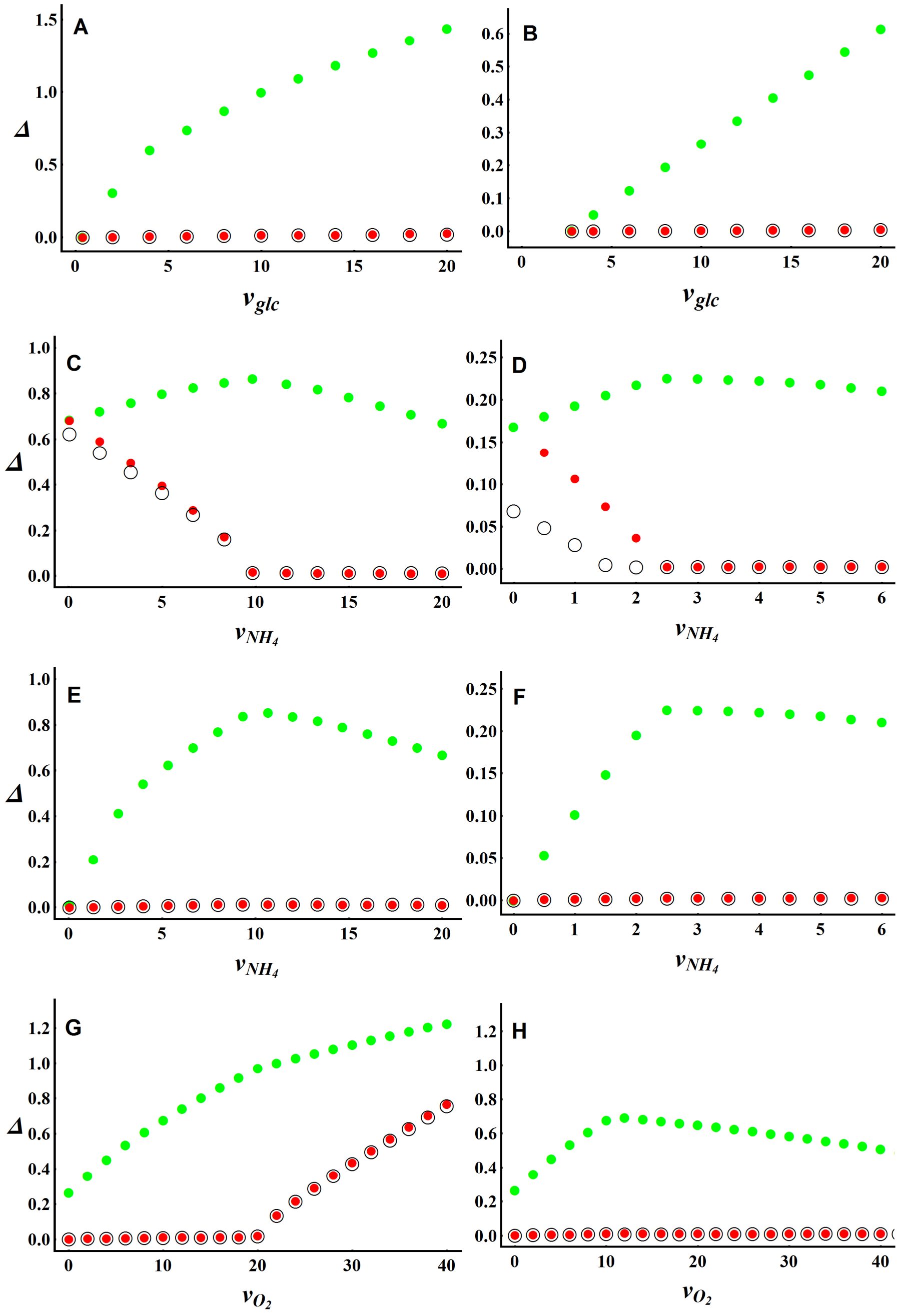

Figure S8. **Flux variability in *iJR904* model.** A, B, C, D, E, F and G, calculated for the *iJR904* model, correspond to Figs. 2, S2, 4A, 4B, 4C, 4D and 3, calculated for the *iJO1366* model, respectively. H differs from G in that it includes the phenomenon of overflow metabolism. For the *iJR904* model, the only significant component of flux variability, in the physiological conditions studied, is growth variability.
